# Mechanistic investigations of antitumor activity of a Rhodamine B-oleanolic acid derivative bioconjugate

**DOI:** 10.3892/or.2021.7981

**Published:** 2021-02-18

**Authors:** Ioana Macașoi, Ioana Zinuca Pavel, Alina Elena Moacă, Ștefana Avram, Vlad Laurențiu David, Dorina Coricovac, Alexandra Mioc, Demetrios A. Spandidos, Aristidis Tsatsakis, Codruța Șoica, Victor Dumitrașcu, Cristina Dehelean

Oncol Rep 44: 1169-1183, 2020; DOI: 10.3892/or.2020.7666

Subsequently to the publication of the above paper, the authors have realized that they should have credited a Professor René Csuk [Martin-Luther-Universität Halle-Wittenberg, Halle (Saale), Germany] for the use of a compound that his group synthesized in the study. Therefore, the authors wish to include the following text in the Acknowledgements section of the Declarations: ‘The authors are grateful to Professor Rene Csuk, Department of Organic Chemistry, Martin-Luther University Halle-Wittenberg, for providing us with the rhodamine B-conjugated oleanolic acid derivative (RhodOA)’. All the named authors agree to this Corrigendum, and apologize to Professor Csuk for the upset and inconvenience caused.

